# Atomic Hydrogen Surrounded by Water Molecules, H(H_2_O)_m_, Modulates Basal and UV-Induced Gene Expressions in Human Skin *In Vivo*

**DOI:** 10.1371/journal.pone.0061696

**Published:** 2013-04-24

**Authors:** Mi Hee Shin, Raeeun Park, Hideo Nojima, Hyung-Chel Kim, Yeon Kyung Kim, Jin Ho Chung

**Affiliations:** 1 Department of Dermatology, Seoul National University College of Medicine, Seoul, Korea; 2 Laboratory of Cutaneous Aging Research, Biomedical Research Institute, Seoul National University Hospital, Seoul, Korea; 3 Institute of Human-Environment Interface Biology, Medical Research Center, Seoul National University, Seoul, Korea; 4 R&D Team, Samsung Electronics CO., LTD, Suwon, Korea; Tohoku University, Japan

## Abstract

Recently, there has been much effort to find effective ingredients which can prevent or retard cutaneous skin aging after topical or systemic use. Here, we investigated the effects of the atomic hydrogen surrounded by water molecules, H(H_2_O)_m_, on acute UV-induced responses and as well as skin aging. Interestingly, we observed that H(H_2_O)_m_ application to human skin prevented UV-induced erythema and DNA damage. And H(H_2_O)_m_ significantly prevented UV-induced MMP-1, COX-2, IL-6 and IL-1β mRNA expressions in human skin *in vivo*. We found that H(H_2_O)_m_ prevented UV-induced ROS generation and inhibited UV-induced MMP-1, COX-2 and IL-6 expressions, and UV-induced JNK and c-Jun phosphorylation in HaCaT cells. Next, we investigated the effects of H(H_2_O)_m_ on intrinsically aged or photoaged skin of elderly subjects. In intrinsically aged skin, H(H_2_O)_m_ application significantly reduced constitutive expressions of MMP-1, IL-6, and IL-1β mRNA. Additionally, H(H_2_O)_m_ significantly increased procollagen mRNA and also decreased MMP-1 and IL-6 mRNA expressions in photoaged facial skin. These results demonstrated that local application of H(H_2_O)_m_ may prevent UV-induced skin inflammation and can modulate intrinsic skin aging and photoaging processes. Therefore, we suggest that modifying the atmospheric gas environment within a room may be a new way to regulate skin functions or skin aging.

## Introduction

Acute exposure to ultraviolet (UV) radiation leads to inflammatory responses such as skin erythema and sunburn, whereas chronic exposure to UV causes carcinogenesis and photoaging of the skin. UV stimulates the expression of a wide variety of proinflammatory mediators, including tumor necrosis factor (TNF)-α, interleukin (IL)-1β, IL-6 and IL-8. It is also known that cyclooxygenase (COX)-2 plays important roles in UV-induced acute inflammation [1], [2], [3], [4], [5]. In fact, inflammation is known to accelerate the aging process, as it is associated with the generation of free radicals and activation of various signaling pathways [6], [7], [8], [9].

Activation of cell surface receptors, such as epidermal growth factor receptor, by UV stimulates mitogen-activated protein kinase (MAPK) signal transduction pathways [10]. Three families of MAPK exist in mammalian cells: extracellular signal-regulated kinase (ERK), c-Jun amino-terminal kinase (JNK) and p38 kinase, and each MAPK form a signaling module. It is reported that MAPKs are involved in the regulation of matrix metalloproteases (MMPs) [11], [12]. MAPKs activation induces both c-Jun and c-Fos, which comprise the transcription factor activator protein (AP)-1 [13], [14]. Many studies have indicated that UV exposure of human skin causes extracellular matrix degradation via induction of transcription factor AP-1 and subsequently by increased MMP production [10], [15]. Another important transcription factor activated in response to UV irradiation is nuclear factor kappa B (NF-κB) [16]. Activation of the NF-κB pathway by UV stimulates inflammatory cytokine expression, and contributes to UV-induced skin damage, such as photoaging.

In addition, human skin is constantly exposed to reactive oxygen species (ROS) from the environment, such as air, solar radiation, ozone and other airborne pollutants, or from the normal metabolism. Accumulated ROS has been suggested to play important roles in the intrinsic aging and photoaging of human skin *in vivo* [17] and is associated with upregulation of MMPs and decreased collagen synthesis [18], [19], [20]. Oxidative stress is thought to play a central role in initiating and driving the signaling events that lead to cellular responses following UV exposure [21], [22], [23], [24]. ROS influences MAPK signaling and thereby contributes to the AP-1-induced up-regulation of MMP-1 [25]. It has been shown that UV-induced ROS production causes skin photoaging and induces the synthesis of MMPs. Thus, strategies to counteract ROS production may be useful for preventing photoaging.

It was found that molecular hydrogen (H_2_) can alleviate ^•^OH-induced cytotoxicity, and that H_2_ has potential as an antioxidant for preventive and therapeutic applications [26]. Actually, inhaled hydrogen molecule gas and hydrogen rich-saline has protective effects on oxidative organ damage, including damaged lung and brain [27], [28], [29], [30]. Inhalation of H_2_ has already been used for the prevention of decompression sickness in divers and has shown a good safety profile [26], [31]. Recently, Nojima et. al reported that atomic hydrogen surrounded by water molecules (H(H_2_O)_m_), released from a novel atmospheric pressure plasma device is effective not only for deactivation of airborne indoor microbial-contaminants but also for neutralization of the OH radicals in the air [32].

In the present study, 1) we demonstrated that local application of H(H_2_O)_n_ gas to human skin could modulate UV-induced skin responses, including sunburn response and DNA damage such as thymidine dimers formation. 2) We also demonstrated that H(H_2_O)_m_ has an anti-oxidant effect and can prevent UV-induced expression of MMP-1, COX-2, IL-6 and IL-1β in human skin *in vivo* and in HaCaT cells. 3) Furthermore, we found that local application of H(H_2_O)_m_ could regulate constitutive expression of genes related to intrinsic aging and photoaging in human skin. 4) Finally, our results indicate that H(H_2_O)_m_ treatment may regulate skin aging process in human skin.

## Results

### H(H_2_O)_m_ treatment prevented UV-induced erythema and thymidine dimers formation in young human skin *in vivo*

**Figure 1:**
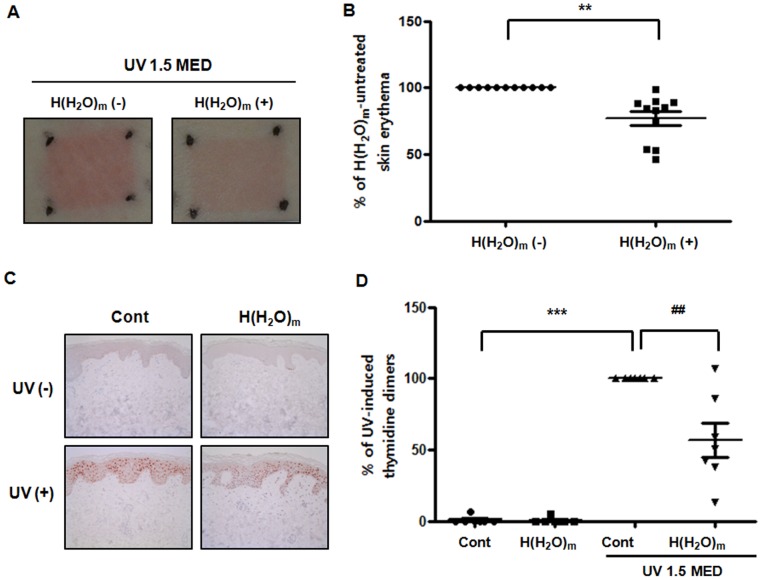
H(H_2_O)_m_ prevents UV-induced erythema and thymidine dimers in young human skin *in vivo*. Skin from young human buttocks was irradiated with UV light and then locally treated with or without H(H_2_O)_m_ for 2 hr. Twenty-four hours after irradiation, erythema index was measured and then skin was biopsied. (**A**) The photographs of erythema are representative of the subjects. (**B**) Erythema-index measurements are shown as means ± SEM with scatter plots (n = 11). (**C**) Immunohistochemical staining was performed using anti-thymidine dimer antibody. The figures shown are representative of seven subjects. (**D**) Results are expressed as means ± SEM with scatter plots (n = 7), *** *p*<0.001 versus the control, ^##^ *p*<0.01 versus UV-irradiated skin.

To investigate whether H(H_2_O)_m_ could prevent UV-induced skin erythema in human skin, the buttocks of young subjects were irradiated with UV (1.5MED), and then treated with H(H_2_O)_m_ for 2 hr. Twenty-four hours after UV irradiation, we observed, interestingly, that UV-induced erythema was reduced in H(H_2_O)_m_-treated skin, compared with control fan-treated skin (Figure 1A). Erythema-index measurements showed that H(H_2_O)_m_ decreased UV-induced erythema by 22.8±5.8%, compared with control skin (Figure 1B). However, we found that UV-induced erythema was not significantly changed by 30 min or 1 hr treatment of H(H_2_O)_m_ (data not shown). These data indicate that H(H_2_O)_m_ has an anti-inflammatory effect against UV-induced sunburn response in human skin. Then, we investigated the effect of H(H_2_O)_m_ on UV-induced DNA damage in human skin *in vivo*. The buttocks of young subjects were irradiated with UV (1.5MED), and then treated with H(H_2_O)_m_ for 2 hr. Twenty-four hours after UV irradiation, we observed UV irradiation of human skin induces DNA damage such as thymidine dimer formation, as shown in figure 1C. Interestingly, local application of H(H_2_O)_m_ significantly decreased UV-induced thymidine dimers formation by 56.7±11.8% compared with UV-irradiated skin (Figure 1C and D).

### H(H_2_O)_m_ treatment prevented UV-induced MMP-1, COX-2, IL-6 and IL-1β in young human skin *in vivo*

**Figure 2:**
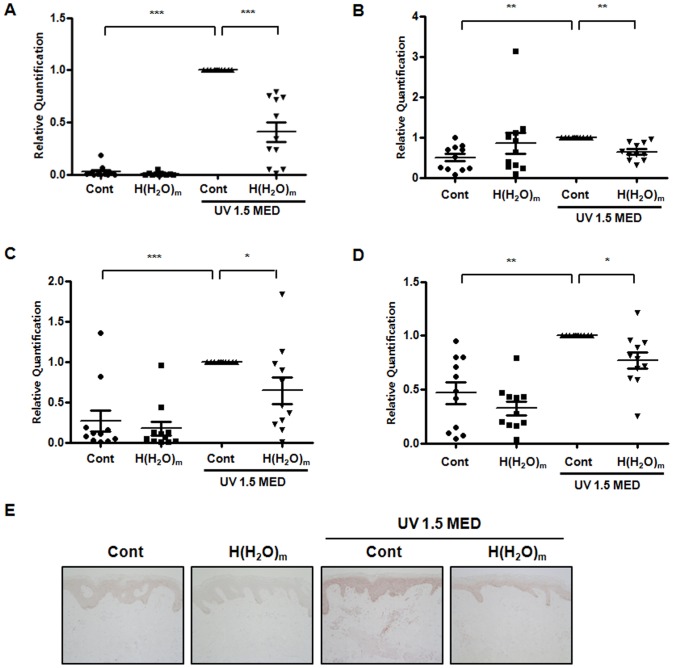
H(H_2_O)_m_ prevents and UV-induced MMP-1, COX-2, IL-6 and IL-1β mRNA expression in young human skin *in vivo*. Skin from young human buttocks was irradiated with UV light and then locally treated with or without H(H_2_O)_m_ for 2 hr. Twenty-four hours after irradiation, and then skin was biopsied. (A) MMP-1, (B) COX-2, (C) IL-6 and (D) IL-1β mRNA expressions were determined by real time RT-PCR. Results are expressed as means ± SEM with scatter plots (n = 11), * *p*<0.05, ** *p*<0.01, *** *p*<0.001. (**E**) Immunohistochemical staining was performed using anti-human MMP-1 antibody. The figures shown are representative of eleven subjects.

Next, by real-time RT-PCR, we demonstrated that H(H_2_O)_m_ prevented UV-induced expressions of MMP-1, COX-2, IL-6 and IL-1β mRNA significantly by 58.9±8.1, 36.1±7.6, 35.4±17.1 and 23.7±9.2%, respectively, compared with UV-irradiated skin (Figure 2A, B, C and D). COX-2 mRNA expression tended to be increased in unirradiated H(H_2_O)_m_, although it was not statistically significant. This tendency seems to be due to unexpected increase of COX-2 mRNA in unirradiated H(H_2_O)_m_ in 2 out of 11 volunteers. However, H(H_2_O)_m_ did not prevent UV-induced decreases in type I procollagen expression (data not shown). Also, similar to UV-induced erythema, we found that UV-induced expression of MMP-1, COX-2, IL-6 and IL-1β mRNA were not significantly changed after 30 min or 1 hr treatments of H(H_2_O)_m_ (data not shown). Immunohistochemical staining revealed that UV induced MMP-1 protein expression throughout the epidermis and that H(H_2_O)_m_ substantially inhibited UV-induced MMP-1 expression versus fan-treated, UV-irradiated skin (Figure 2E). These results suggest that H(H_2_O)_m_ may prevent acute UV-induced skin responses.

### H(H_2_O)_m_ prevented UV-induced MMP-1, COX-2 and IL-6 expressions, and inhibited UV-induced SEK1/JNK activation and c-Jun phosphorylation in HaCaT cells

**Figure 3:**
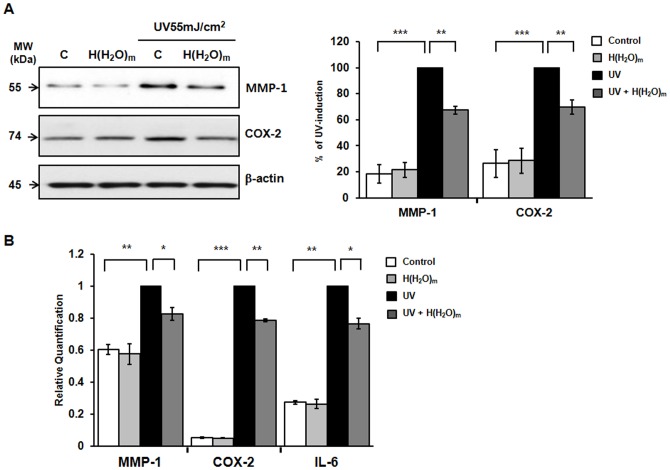
H(H_2_O)_m_ prevents UV-induced MMP-1, COX-2 and IL-6 mRNA expression in HaCaT cells. (**A**) Cells were pre-treated with H(H_2_O)_m_ for 15 min, then irradiated with UV (55 mJ/cm^2^) and post-treated with H(H_2_O)_m_ for 15 min in PBS. After incubation for 48 hr with serum-free DMEM, MMP-1 expression was determined in culture media and COX-2 and β-actin expression were determined in cell lysates by Western blotting. The bands are representative of results from three independent experiments. Results are expressed as means ± SEM (n = 6). (**B**) Cells were treated with H(H_2_O)_m_ and UV as described above. After incubation for 24 hr with serum free DMEM, MMP-1, COX-2 and IL-6 mRNA expressions were determined by real-time RT-PCR. Results are expressed as means ± SEM (n = 3), * *p*<0.05, ** *p*<0.01, *** *p*<0.001.

To confirm and investigate the action mechanisms of H(H_2_O)_m_ on UV-induced skin inflammation in human skin *in vivo*, we used HaCaT cells for the next experiments. Cells were irradiated with 55 mJ/cm^2^ of UV with or without H(H_2_O)_m_ treatment. By western blotting, we demonstrated that H(H_2_O)_m_ significantly prevented up-regulation of MMP-1 and COX-2 expression by UV (Figure 3A). Real-time PCR reveals that UV-induced MMP-1, COX-2 and IL-6 mRNA expression was inhibited by H(H_2_O)_m_ in a pattern similar to the Western blotting results (Figure 3B). From these results, we demonstrated that H(H_2_O)_m_ decreased the UV-induced MMP-1, COX-2 and IL-6 in human keratinocyte.

**Figure 4:**
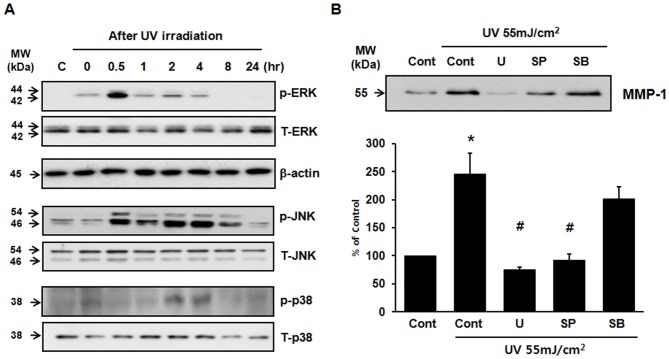
UV-induced MMP-1 expression is mediated by ERK and JNK activation but not by p38 activation in HaCaT cells. (**A**) Cells were serum-starved for 24 hr, irradiated with UV light (55 mJ/cm^2^) and harvested at the indicated times. Total cell lysates were prepared and subjected to Western blotting using specific antibodies. The bands are representative of results from three independent experiments. (**B**) HaCaT cells were pretreated with U0126(U) [ERK (MEK1) inhibitor; 10 µM], SP600125(SP) [JNK inhibitor; 10 µM], and SB203580(SB) [p38 inhibitor; 10 µM] for 1 hr, and then irradiated with UV. Irradiated cells were cultured for 48 hr and MMP-1 expression was determined by Western blotting. All experiments were performed in triplicate. Values shown are means ± SEM (n = 4), * *p*<0.05 versus the control, ^#^ *p*<0.05 versus UV-irradiated cells.

In HaCaT cells, MAPKs were activated by UV within 30 min after UV irradiation and then declined to basal levels (Figure 4A). To investigate the roles of MAPKs in UV-induced MMP-1 expression, cells were pretreated with U0126 (10 µM; MEK1-inhibitor), SP600125 (10 µM; JNK-inhibitor), or SB203580 (10 µM; p38-inhibitor) for 1 hr and then irradiated with UV. UV-induced MMP-1 expression was inhibited by pretreating with U0126 and SP600125, but not by SB203580 (Figure 4B). These results suggest that the activation of the ERK and JNK pathways mediates UV-induced MMP-1 expression in HaCaT cells.

**Figure 5:**
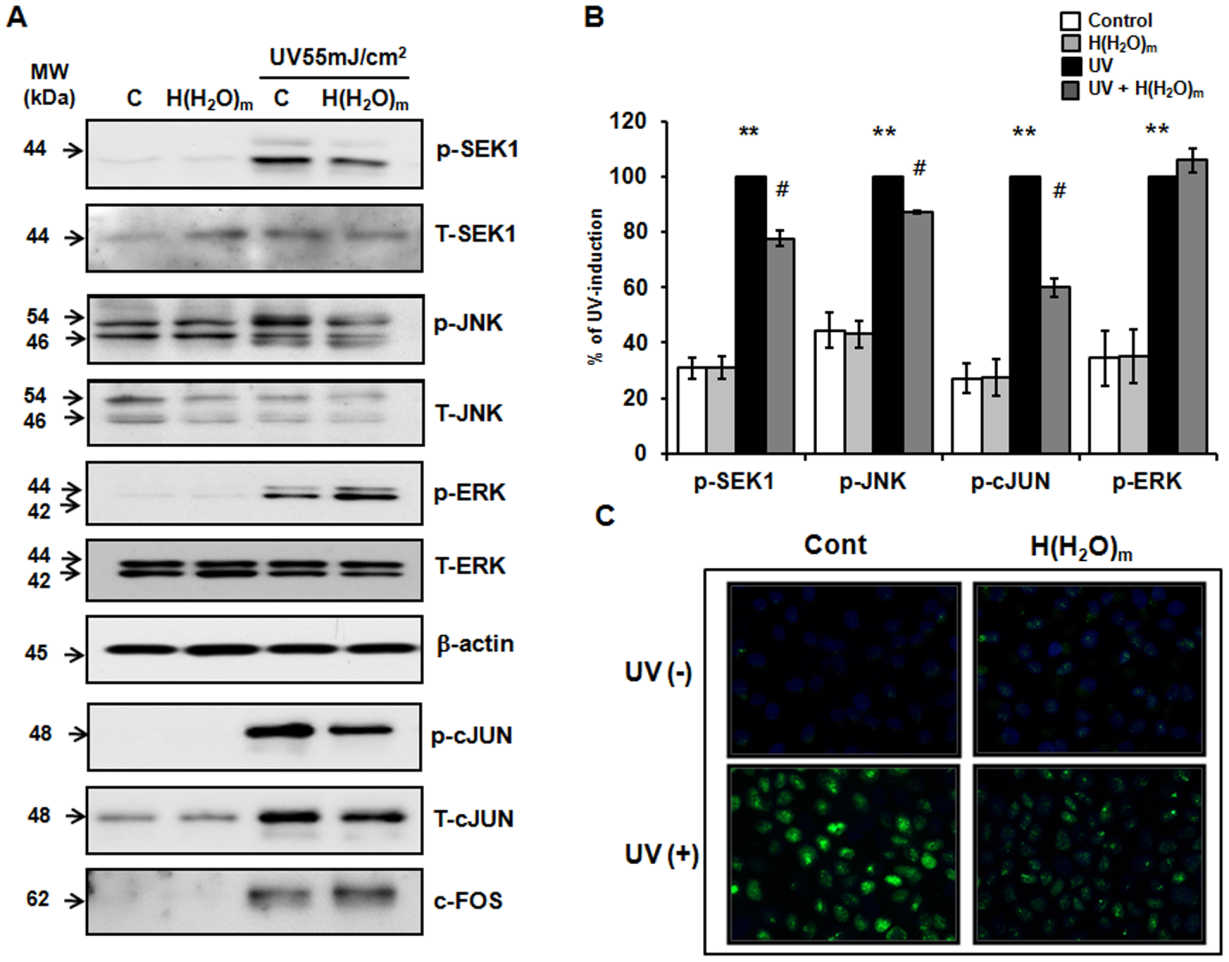
UV-induced SEK1/JNK activation and c-Jun phosphorylation are inhibited by H(H_2_O)_m_ treatment in HaCaT cells. (**A**) Cells were pre-treated with H(H_2_O)_m_ for 15 min, then irradiated with UV and post-treated with H(H_2_O)_m_ for 15 min in PBS. After incubation for 30 min, total cell lysates were prepared. Western blotting were performed using phospho-specific (p−) and total (T−) JNK, c-Jun, SEK1 and ERK antibodies. Level of β-actin was used as loading control. The bands are representative of results from three independent experiments. (**B**) Results are expressed as means ± SEM (n = 3), ** *p*<0.01 versus the control, ^#^ *p*<0.05 versus UV-irradiated cells. (**C**) Intracellular phospho-c-Jun levels in cells were visualized using a fluorescent microscope and the images presented are representative of the fluorescence levels observed in three separate experiments.

As ERK and JNK activation are required for UV-induced MMP-1 expression in HaCaT cells, we investigated the effects of H(H_2_O)_m_ on UV-induced ERK and JNK activation. Treatment with H(H_2_O)_m_ significantly inhibited JNK phosphorylation without altering total JNK levels (Figure 5A and B). But, UV-induced ERK activation was not inhibited by H(H_2_O)_m_ treatment. We also found that H(H_2_O)_m_ significantly inhibited the UV-induced phosphorylation of SEK1, which is the upstream kinase of JNK (Figure 5A and B). These results suggest that inhibition of SEK1/JNK pathways by H(H_2_O)_m_ might attenuate UV-induced MMP expression.

AP-1 is known to play a critical role in MMP-1 expression by UV [33], [34], [35]. The transcriptional activity of AP-1 is also dependent on the degree of phosphorylation of c-Jun. Therefore, we investigated the effect of H(H_2_O)_m_ on UV-induced c-Jun phosphorylation in HaCaT cells. UV was found to increase the level of phosphorylated c-Jun and H(H_2_O)_m_ inhibited UV-induced phospho-c-Jun expression significantly (Figure 5A and B). In addition, although UV irradiation increased the expression of c-Fos, H(H_2_O)_m_ did not inhibit UV-induced c-Fos expression. In the result of immunofluorescence staining, UV-induced phospho-c-Jun expression was decreased by H(H_2_O)_m_ (Figure 5C). These results suggest that H(H_2_O)_m_ inhibits UV-induced MMP-1 expression and that this inhibition may be mediated by a reduction in the level of phosphorylated c-Jun, which is known to be closely associated with UV-induced AP-1 activation. Because JNK is closely related to c-Jun phosphorylation and expression, these findings suggest that the inhibition of UV-induced phospho-cJun by H(H_2_O)_m_ may be mediated by inhibition of the activation of JNK.

### H(H_2_O)_m_ reduced UV-induced ROS generation in HaCaT cells

**Figure 6:**
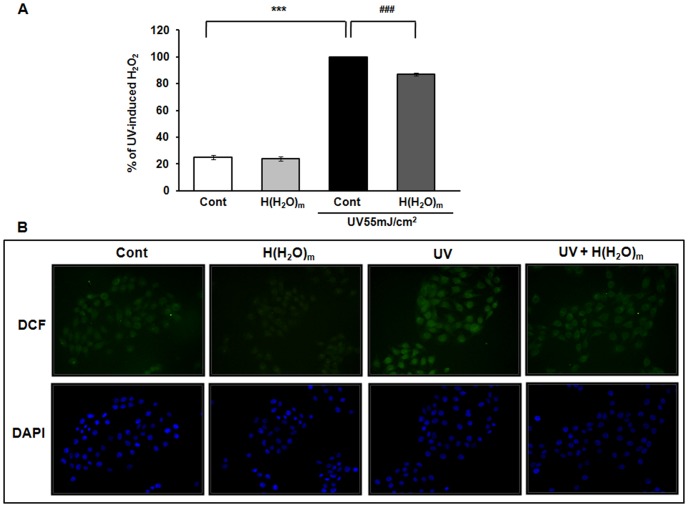
H(H_2_O)_m_ reduces UV-induced ROS production in HaCaT cells. (**A**) Cells were pre-treated with H(H_2_O)_m_ for 15 min, then irradiated with UV and post-treated with H(H_2_O)_m_ for 15 min in PBS. Subsequently, cells were treated with DCFDA (25 µM, freshly diluted in pre-warmed DMEM) for 30 min. Cells were then assayed using a fluorescence reader. Values shown are means ± SEM (n = 8), *** *p*<0.001 versus the control, ^###^ *p*<0.001 versus UV-treated cells. (**B**) Intracellular H_2_O_2_ levels in HaCaT cells were visualized after DCF staining. The fluorescence intensity was visualized using a fluorescent microscope, and the images presented are representative of the fluorescence levels observed in three separate experiments. To normalize cell number, DAPI was used as a fluorescent marker for the nucleus.

UV causes generation of ROS and UV-triggered oxidative stress regulates a variety of cellular functions including MMP secretion and cytokine production [36], [37], [38], [39]. It was proposed that antioxidants scavenging ROS can prevent skin photoaging. Therefore, we investigated whether H(H_2_O)_m_ may have an antioxidant effect. UV significantly increased ROS generation by 409.4±21.1% and H(H_2_O)_m_ significantly reduced this UV-induced ROS generation by 12.9±1.1%, compared with the UV-treated cells (Figure 6A). Also, in the result of DCF staining, we confirmed that H(H_2_O)_m_ has ROS scavenging effect against UV-induced ROS (Figure 6B).

### H(H_2_O)_m_ decreased basal expressions of MMP-1, IL-6 and IL-1β in intrinsically aged human skin

**Figure 7:**
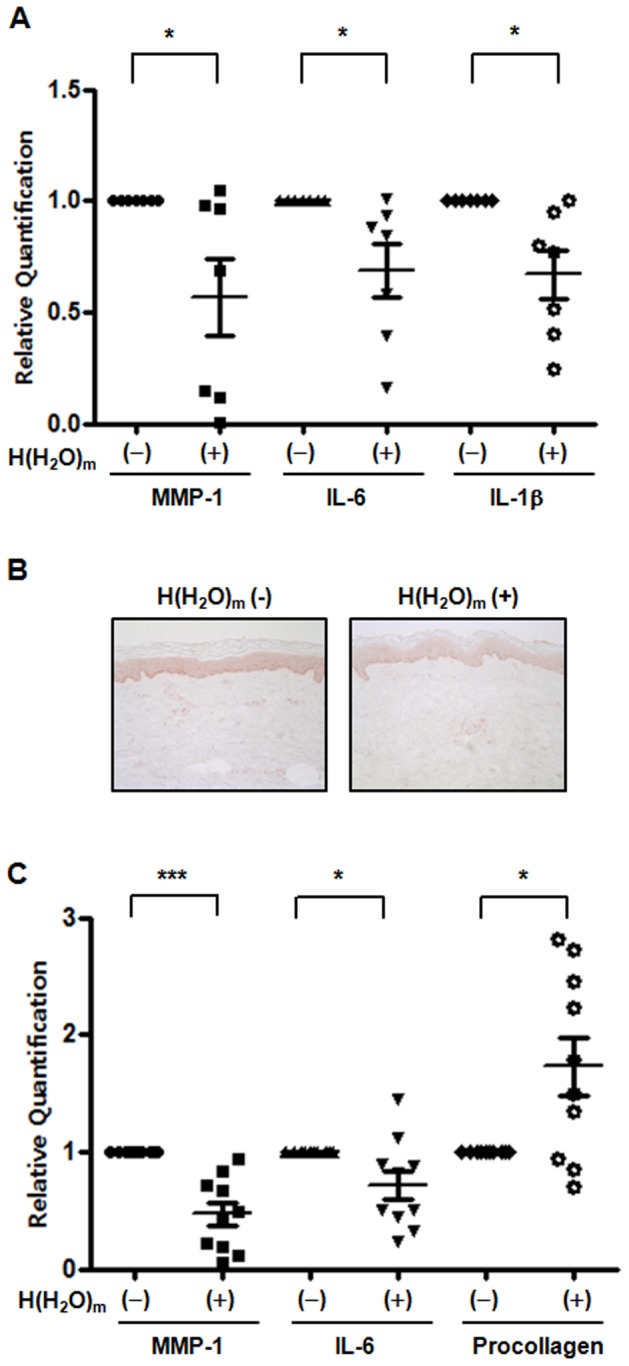
Basal expression of MMP-1, COX-2 and IL-6 are decreased by H(H_2_O)_m_ treatment in intrinsically aged human buttock skin and H(H_2_O)_m_ increases basal expression of type I procollagen expression in photoaged facial skin and also reduces the basal expressions of MMP-1 and IL-6. (**A**) Aged human buttock skin was topically treated with or without H(H_2_O)_m_ using a releasing device for 2 hr. MMP-1, COX-2 and IL-6 mRNA expressions were determined by real time RT-PCR. Results are shown as means ± SEM with scatter plots (n = 7), * *p*<0.05. (**B**) Immunohistochemical staining was performed using anti-human MMP-1 antibody. The figures shown are representative of seven subjects. (**C**) Aged human face skin was topically treated with or without H(H_2_O)_m_ for 30 min a day for 4 days. Type I procollagen, MMP-1 and IL-6 mRNA expressions were determined by real time RT-PCR. Results are expressed as means ± SEM with scatter plots (n = 10), * *p*<0.05, *** *p*<0.001.

Next, we investigated whether H(H_2_O)_m_ may have beneficial effects on intrinsically aged skin of the elderly subjects. The aged human buttock skin was treated with H(H_2_O)_m_ for 2 hr, and then skin was biopsied 24 hr post-treatment. By real-time RT-PCR analysis, we demonstrated that H(H_2_O)_m_ reduced the constitutive expressions of MMP-1, IL-6 and IL-1β mRNA, significantly (Figure 7A). However, the basal expression of type I procollagen mRNA was not changed by H(H_2_O)_m_ in aged skin. In addition, by immunohistochemical staining, we found that the basal level of MMP-1 protein expression in the aged skin was decreased by H(H_2_O)_m_ (Figure 7B).

### H(H_2_O)_m_ increased basal expression of type I procollagen in photoaged facial skin and also reduced the basal expressions of MMP-1 and IL-6

Photoaged facial skin was treated with H(H_2_O)_m_ for 30 min/day for four consecutive days sequentially, and then 24 hr after the last treatment, skin was biopsied. Similar to data on intrinsic aging, MMP-1 and IL-6 mRNA levels were significantly decreased by 52.3±10.2 and 27.8±12.7%, respectively, by H(H_2_O)_m_ (Figure 7C). However, interestingly, in contrast to intrinsically aged skin, H(H_2_O)_m_ significantly increased basal expression of type I procollagen mRNA by 166.3±28.1%, compared with control fan-treated facial skin (Figure 7C). These results suggest that H(H_2_O)_m_ may modulate basal gene expression in aged and photoaged human skin.

## Discussion

This study demonstrated that H(H_2_O)_m_ prevented UV-induced erythema and DNA damage in human skin and also inhibited UV-induced MMP-1, COX-2, IL-6 and IL-1β expressions significantly. Also, we found that H(H_2_O)_m_ exposure to human skin could modulate basal mRNA expressions of MMP-1, type I procollagen, COX-2, IL-1β and IL-6 in intrinsically aged and photoaged human skin. To our knowledge, this is the first report that an atmospheric gas component, such as H(H_2_O)_m_, could modulate UV-induced skin responses and regulate basal gene expression in aged human skin. Since there are some reports that gas or air can penetrate or pass through the skin [40], [41], we speculated that H(H_2_O)_m_ may penetrate the skin barrier and subsequently affect skin cells functions through an unknown mechanism.

UV-induced erythema is the most obvious of the photobiological responses evidenced by skin and a marker of tissue injury and inflammation [42], [43]. DNA damage by UV exposure plays an essential part, and is the initial step, in skin cancer induction [44], [45]. In our study, we determined that treatment of H(H_2_O)_m_ suppressed significantly UV-induced erythema response in human skin *in vivo*, and UV-induced thymidine dimer formation was also inhibited by H(H_2_O)_m_ in human skin. Moreover, the sunburn-reducing effect and DNA damage-inhibiting effect of H(H_2_O)_m_ may be in part due to its anti-inflammatory and anti-oxidant effects, based on the our results that H(H_2_O)_m_ inhibited UV-induced expression of COX-2 and inflammatory cytokines, including IL-6 and IL-1β, and reduced UV-induced ROS generation. Taken together, we propose that local application of H(H_2_O)_m_ can protect our skin from UV-induced inflammatory responses and DNA damages.

To understand the changes of signaling pathways caused by H(H_2_O)_m_ treatment leading to decreased expression of MMP-1 expression, we performed the experiments using human keratinocytes, HaCaT cells. MAPK is known to play an essential role in induction of MMP-1 expression by UV [46], [47]. MAPKs activation is followed by an increase in the expression of c-Jun and c-Fos, which form the AP-1 complex. Transcription of several MMPs, including MMP-1, MMP-3 and MMP-9, is regulated by AP-1 [12], which is one of several transcriptional factors activated by UV [48]. Thus, increased AP-1 activity is responsible for the degradation of extracellular matrix proteins, such as collagen, by inducing MMPs. In human skin, UV-induced AP-1 transcriptional activity is determined by c-Jun expression, because c-Fos is expressed continuously [12], [49]. Whereas c-Fos expression in young and aged skin is unaltered, c-Jun expression is higher in aged skin than in young skin [14]. In this study, we found that H(H_2_O)_m_ treatment inhibited UV-induced c-Jun phosphorylation, but c-Fos expression was not suppressed by H(H_2_O)_m_. Our results demonstrated that H(H_2_O)_m_ treatment inhibits UV-induced MMP-1 and that this inhibition may be mediated by a reduction in the level of phosphorylated SEK1/JNK and c-Jun, which are known to be closely associated with UV-induced AP-1 activation in HaCaT cells.

On the other hand, UV is also known to activate NF-κB and regulate downstream NF-κB-dependent genes such as COX-2 and inflammatory cytokines [50], [51]. In this study, H(H_2_O)_m_ treatment suppressed the expressions of UV-induced COX-2 and IL-6, but H(H_2_O)_m_ did not inhibit UV-induced NF-κB activation (data not shown). Therefore, our results suggest that the inhibition of UV-induced COX-2 and IL-6 by H(H_2_O)_m_ is not associated with NF-κB, and the exact action mechanism of H(H_2_O)_m_ remains to be investigated.

UV irradiation initiates the generation of ROS, which causes MMP-1 upregulation and the degradation of dermal collagen, possibly by activating the MAPK signaling pathway [52]. This mechanism predicts that a free radical scavenger might prevent UV-induced skin damage by inhibiting the induction of MMPs. Also, in the previous study, significant protective effects against OH radicals by H(H_2_O)_m_ in the air was observed in mammalian cells [32]. Cell death induced by an OH generator was suppressed by H(H_2_O)_m_ treatment [32]. In this study, we also found that H(H_2_O)_m_ inhibits UV-induced H_2_O_2_ generation, suggesting that the inhibition of UV-induced MMP-1 expression by H(H_2_O)_m_ may be associated with the inhibition of ROS production due to antioxidant effects of H(H_2_O)_m_ in HaCaT cells.

Although the typical appearance of photoaged and chronologically aged human skin can be readily distinguished, it has been reported that intrinsically aged and photoaged skin share important molecular features such as altered signal transduction pathways that promote MMP expression as well as decrease procollagen synthesis. This concordance of molecular mechanisms suggests that UV irradiation may accelerate many key aspects of the aging process in human skin [53], [54]. In many studies, researchers have tried to develop new agents for the prevention of skin aging. The approaches to improve UV-induced skin damage in the aged skin could involve the use of antioxidant agents or potential sources of MMP-1 inhibitors and collagen-synthesis inducers, in addition to the administration of anti-inflammatory agents. Interestingly, this study demonstrated that H(H_2_O)_m_ treatment decreased basal MMP-1, COX-2, IL-6 mRNA expressions in intrinsically aged and photoaged human skin *in vivo*. In contrast to intrinsically aged skin, H(H_2_O)_m_ treatment increased basal expression of type I procollagen mRNA in photoaged facial skin. Therefore, we suggest that applying H(H_2_O)_m_ to the skin could be a new way to prevent UV-induced skin damage and slow aging of the skin. We also speculate that regulating atmospheric gas composition in a room or office may be an effective strategy to modulate skin functions.

## Materials and Methods

### Device Design

The developed device consists of a ceramic plate and a needle-shaped electrode with inner and outer electrodes as described previously [32]. When a pulse-shaped voltage is applied between the inner and outer electrodes of the plate, H^+^ surrounded by water molecules, H^+^(H_2_O)_m_ is generated as a positive ion (Figure S1, S2, S3). H^+^(H_2_O)_m_ was measured by ion counter. It measured about 1∼2×10^6^ ions/cm^3^ at 0.3 m distance. Then, the positive ion, H^+^(H_2_O)_m_ is neutralized after combining with electrons from needle-shaped electrode and becomes atomic hydrogen surrounded by water molecules, H(H_2_O)_m_. H^+^(H_2_O)_m_ after combining with electrons from needle-shaped electrode was less than 1×10^4^ ions/cm^3^, which is known to be normal level on the atmospheric pressure. This means most H^+^(H_2_O)_m_ may be converted to H(H_2_O)_m_. The generation of atomic hydrogen in the device was detected by an optical spectroscopic method [32]. This device was manufactured by Samsung Electronics for the cell experiments and local application on human skin. The schematic experimental designs were showed in supporting information (Figure S1, S2, S3).

### Cell culture, UV irradiation and H(H_2_O)_m_ treatment

Immortalized human keratinocyte HaCaT cells were grown in Dulbecco's modified eagle medium (DMEM) supplemented with 10% heat-inactivated fetal bovine serum (FBS), 2 mM glutamine, penicillin (100 U/ml) and streptomycin (100 µg/ml) in a 37°C humidified 5% CO_2_ incubator. In all experiments, cells were cultured to 80% confluence and then starved in serum-free DMEM for 24 hr.

For cell experiments, the device was made as shown in figure S1. Briefly, the device releasing hydrogen atom (H(H_2_O)_m_) was installed in the top of a chamber. A fan was also installed in the chamber to aid the dispersion and circulation of the device-generated H(H_2_O)_m_. For control treatment, only a fan was installed in the chamber. The cells were pre-treated with H(H_2_O)_m_ for 15 min then irradiated with UV 55 mJ/cm^2^ and post-treated with H(H_2_O)_m_ for 15 min in PBS. Philips TL20W/12RS fluorescent sun lamps (Einthoven, Netherlands) with an emission spectrum between 275 and 380 nm were used as the UV source [55]. The distribution of the power output of the lamps was 0.5% UVC (<280 nm), 56.7% UVB (280–320 nm), and 42.8% UVA (320–400 nm). And a Kodacel filter (TA401/407; Kodak, Rochester, NY) was used to remove wavelengths <290 nm (UVC). Therefore, in this experiment, used UV light includes UVA plus UVB, which we just commonly referred to as “UV”. The UV strength was measured using a UV meter (Model 585100; Waldmann, Villingen-Schwenningen, Germany).

### Local treatment of H(H_2_O)_m_ to human skin and UV irradiation

To investigate the effects of H(H_2_O)_m_ treatment on UV-induced skin damage in human skin *in vivo*, eleven young Koreans (mean age, 31.8 yr; age range, 24–47 yr), were irradiated by UV (1.5 MED). For human experiment, the device was made as shown in figure S2 and S3. Minimal erythema dose (MED) for each subject was determined 24 hr after irradiation of the buttock skin. MED ranged between 70 and 90 mJ/cm^2^ for the skin of Koreans. The buttock skin was irradiated with UV and subsequently, treated with H(H_2_O)_m_ for 2 hr. Twenty-four hours post-irradiation, we measured UV-induced erythema using a DermaSpectrometer® (Cortex Technology, Hadsund, Denmark) and buttock skin was biopsied.

To investigate the effects of H(H_2_O)_m_ on intrinsically aged skin, buttock skin of the elderly subjects (seven Koreans; mean age, 73.1 yr; age range, 65–81 yr) were treated with H(H_2_O)_m_ for 2 hr. Twenty-four hours after H(H_2_O)_m_ treatment, buttock skin was biopsied.

To evaluate the effects of H(H_2_O)_m_ on the photoaged skin, photoaged facial skin (crow's feet area) of the elderly subjects (ten Koreans; mean age, 54.1 yr; age range, 45–62 yr) was topically treated with H(H_2_O)_m_ for 30 min a day for sequential four days. For control treatment, the other side of each photoaged facial skin of the volunteers was treated with only a fan. Twenty-four hours after the last treatment, facial skin was biopsied for real-time RT-PCR analysis. This study was conducted according to the Declaration of Helsinki Principles. All procedures received prior approval from the Institutional Review Board at Seoul National University Hospital and all subjects gave written informed consent.

### Western blot analysis

To determine the amount of MMP-1 secreted into the culture media, equal aliquots of conditioned culture media from an equal number of cells were fractionated by 10% SDS PAGE, transferred to a Hybond ECL membrane (Amersham Biosciences, Buckinghamshire, England) and analyzed by Western blotting with a antibody against MMP-1 (Lab Frontier, Seoul, Korea) using enhanced chemiluminescence (Amersham Biosciences). To analyze MAPK activation, total cell lysates were prepared in a lysis buffer [25 mM Hepes (pH 7.7), 0.3 M NaCl, 1.5 mM MgCl_2_, 0.2 mM ethylenediamine tetraacetic acid (EDTA), 0.1% Triton X-100, 0.5 mM dithiothreitol (DTT), 20 mM β-glycerolphosphate, 0.1 mM Na_3_VO_4_, 2 µg/ml leupeptin, 2 µg/ml aprotinin, 1 mM phenylmethylsulfonyl fluoride (PMSF), and a protease inhibitor cocktail tablet from Boehringer Mannheim (Indianapolis, IN)]. The protein concentrations were determined by Bradford assay using Bio-Rad Protein Assay reagents (Bio-Rad Laboratories, Hercules, CA). Equal amounts of protein samples were fractionated and transferred as described above and analyzed by Western blotting using phospho-specific antibodies against ERK1/2, JNK, and p38 MAPK (Cell Signaling Technology, Beverly, MA). As controls, the levels of the corresponding total MAPK were determined in the same samples using specific antibodies for ERK1/2, JNK and p38 MAPK (Cell Signaling Technology). Blotting proteins were visualized by enhanced chemiluminescence (Amersham, Buckinghamshire, England) and exposed to Kodak X-ray film. The band intensities were measured using Bio 1D software (Vilber Lourmat, Marne La Vallec, France).

### Quantitative real-time RT-PCR

Total RNA was isolated using Trizol (Invitrogen, Carlsbad, CA) and 1 µg of total RNA was converted to cDNA using a First Strand cDNA Synthesis Kit (MBI Fermentas, Vilnius, Lithuania) according to the manufacturer's instructions. Quantitation of MMP-1, procollagen, COX-2, COX-1, IL-6, IL-1β and endogenous reference 36B4 cDNA was performed using a 7500 Real-time PCR System (Applied Biosystems, Foster City, CA). Data were analyzed using the 2(−Delta Delta C[T]) methods [56]. Data are expressed as the means ± SEM normalized to 36B4 and relative to the control sample. These experiments were carried out in triplicate.

### Immunohistochemical staining

The frozen sections of 4 µm thickness were mounted onto silane coated slides (Dako, Glostrup, Denmark), then rehydrated in DW and endogenous peroxidase activity was quenched using 3% hydrogen peroxide for 10 min. The sections were then blocked with blocking solution (Zymed, San Francisco, CA) for 30 min, and washed and incubated with primary antibodies [anti-thymidine dimer antibody (Kamiya Co., Seattle, WA) or anti-MMP-1 antibody (R&D Systems, Minneapolis, MN)] in a humidified chamber at 4°C for 18 hr. After washing in PBS, they were incubated with biotinylated secondary antibody for 30 min, followed by horseradish-streptoavidin conjugate for 15 min. After a further washing in PBS, color was developed using AEC (3-amino-9-ethylcarbazole; Zymed).

### Measurement of intracellular H_2_O_2_

Intracellular H_2_O_2_ levels were determined by measuring 2,7-dichlororofluorescein diacetate (DCFDA, Molecular Probes, Eugene, Oregon) fluorescence. HaCaT cells were cultured in DMEM until 80% confluent in 24-well plates, and starved with serum free DMEM for 24 h. Cells were pre-treated with H(H_2_O)_m_ m for 15 min, then irradiated with UV (55 mJ/cm^2^) and post-treated with H(H_2_O)_m_ for 15 min in PBS. Subsequently, cells were treated with DCFDA (25 µM, freshly diluted in pre-warmed DMEM) for 30 min. Cells were then assayed using a fluorescence reader at an excitation wavelength of 488 nm and an emission wavelength of 515 nm. The fluorescence for DCF staining was detected at 485 nm/515 nm using a fluorescent microscope (Venox AHBT3/Q imaging system, Olympus, Tokyo, Japan). To normalize cell number, 4, 6-diamidino-2-phenylindole dihydrochloride hydrate (DAPI: 1 µg/ml, Molecular Probes) was used as a fluorescent marker for the nucleus with an excitation wavelength of 364 nm and an emission wavelength of 480 nm under a fluorescent microscope. The experiments were repeated at least three times per treatment.

### Statistical analysis

Statistical analyses were performed using a Student's t-test. *p*-values of less than 0.05 were considered statistically significant.

## Supporting Information

Figure S1 **Schematic experimental design for the cell experiment.**
(TIF)

Figure S2 **Schematic experimental design for local application in human buttock skin.**
(TIF)

Figure S3 **Schematic experimental design for local application in aged human facial skin.**
(TIF)
