# Supplementary figures and images for: Atomic Hydrogen Surrounded by Water Molecules, H(H2O)m, Modulates Basal and UV-Induced Gene Expressions in Human Skin In Vivo
Source: PLoS One. 2013 Apr 24;8(4):e61696. doi: 10.1371/journal.pone.0061696 (PMC3634861; doi:10.1371/journal.pone.0061696)

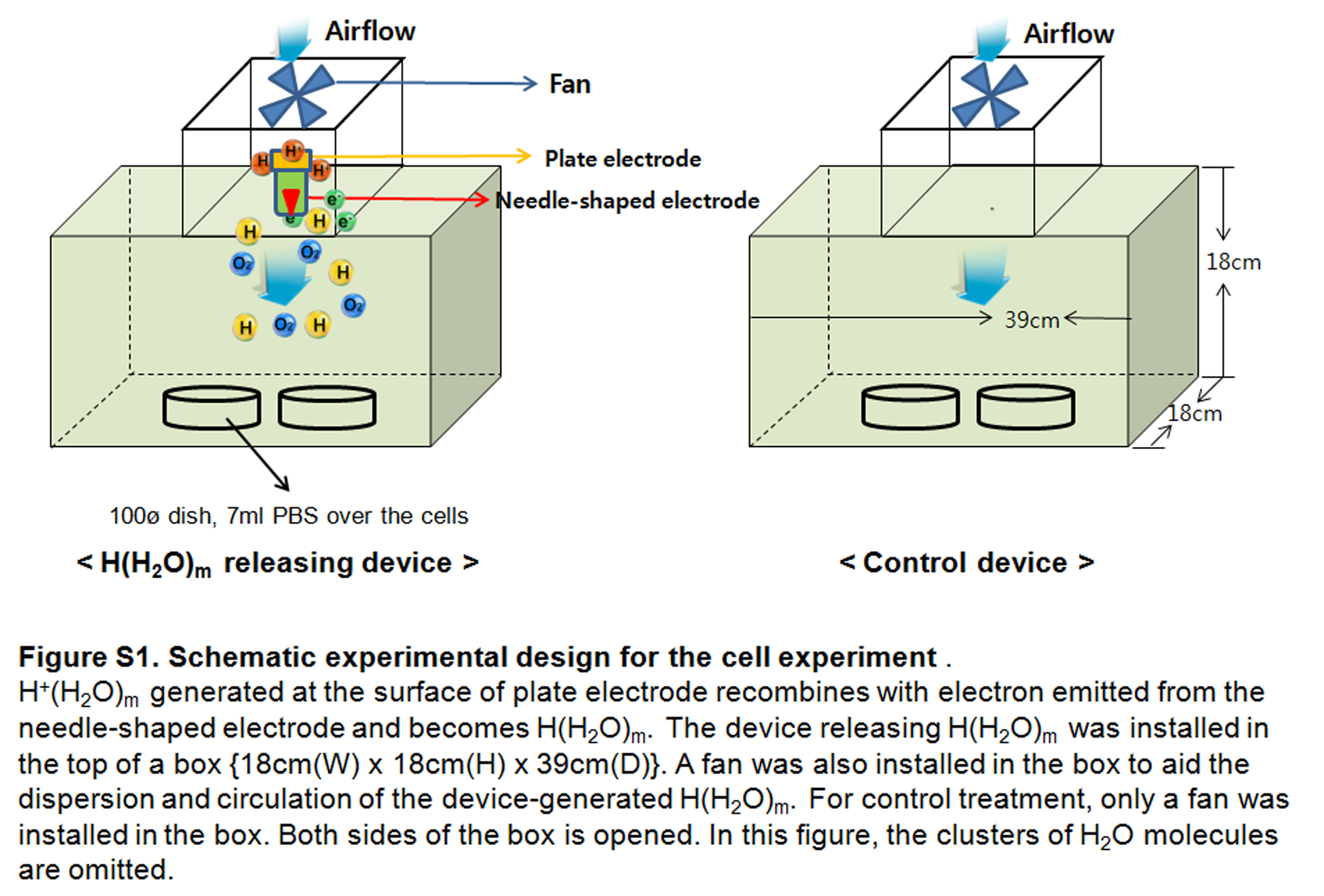

Supplement: Figure S1 — Schematic experimental design for the cell experiment. (TIF) [file pone.0061696.s001.tif]

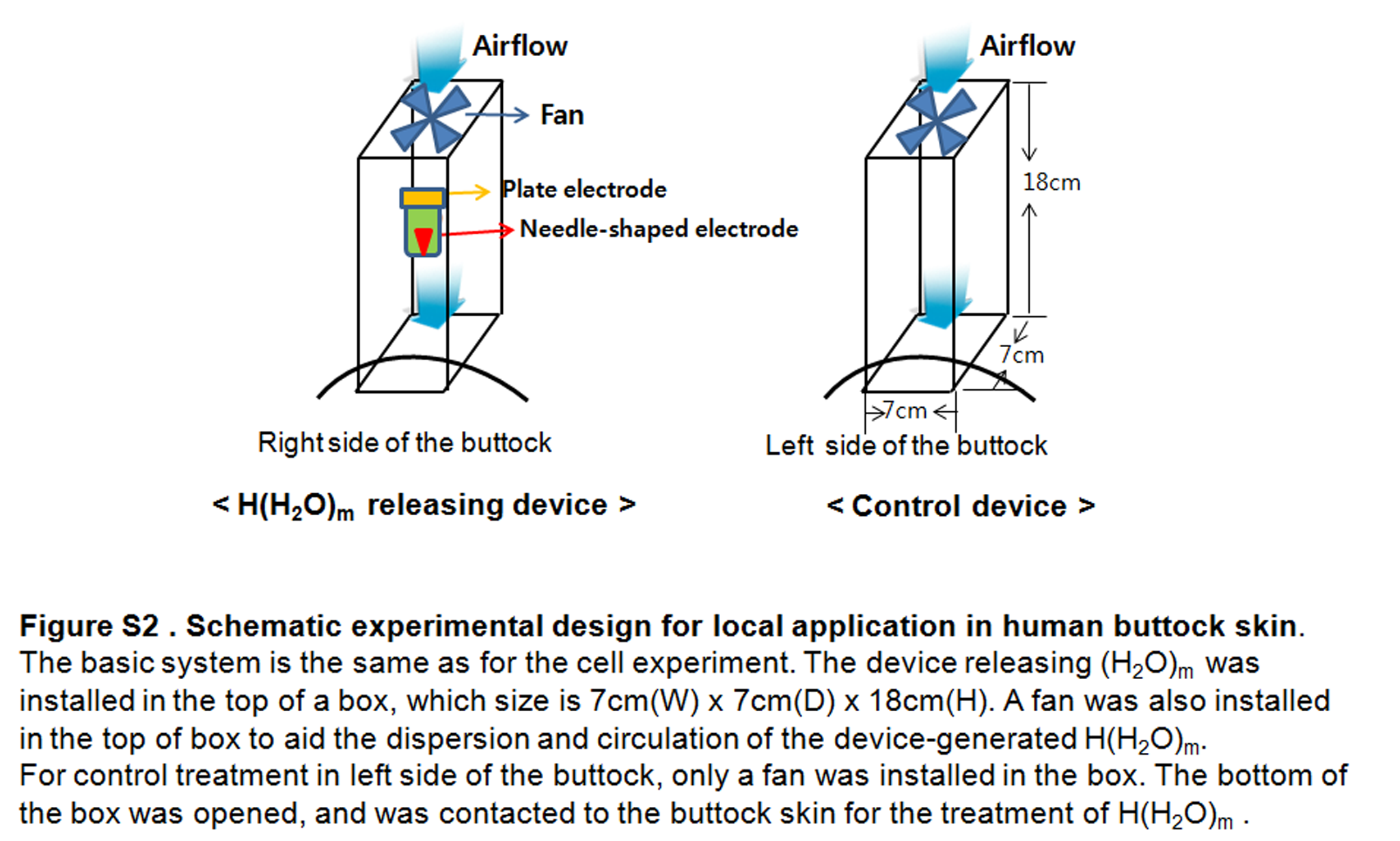

Supplement: Figure S2 — Schematic experimental design for local application in human buttock skin. (TIF) [file pone.0061696.s002.tif]

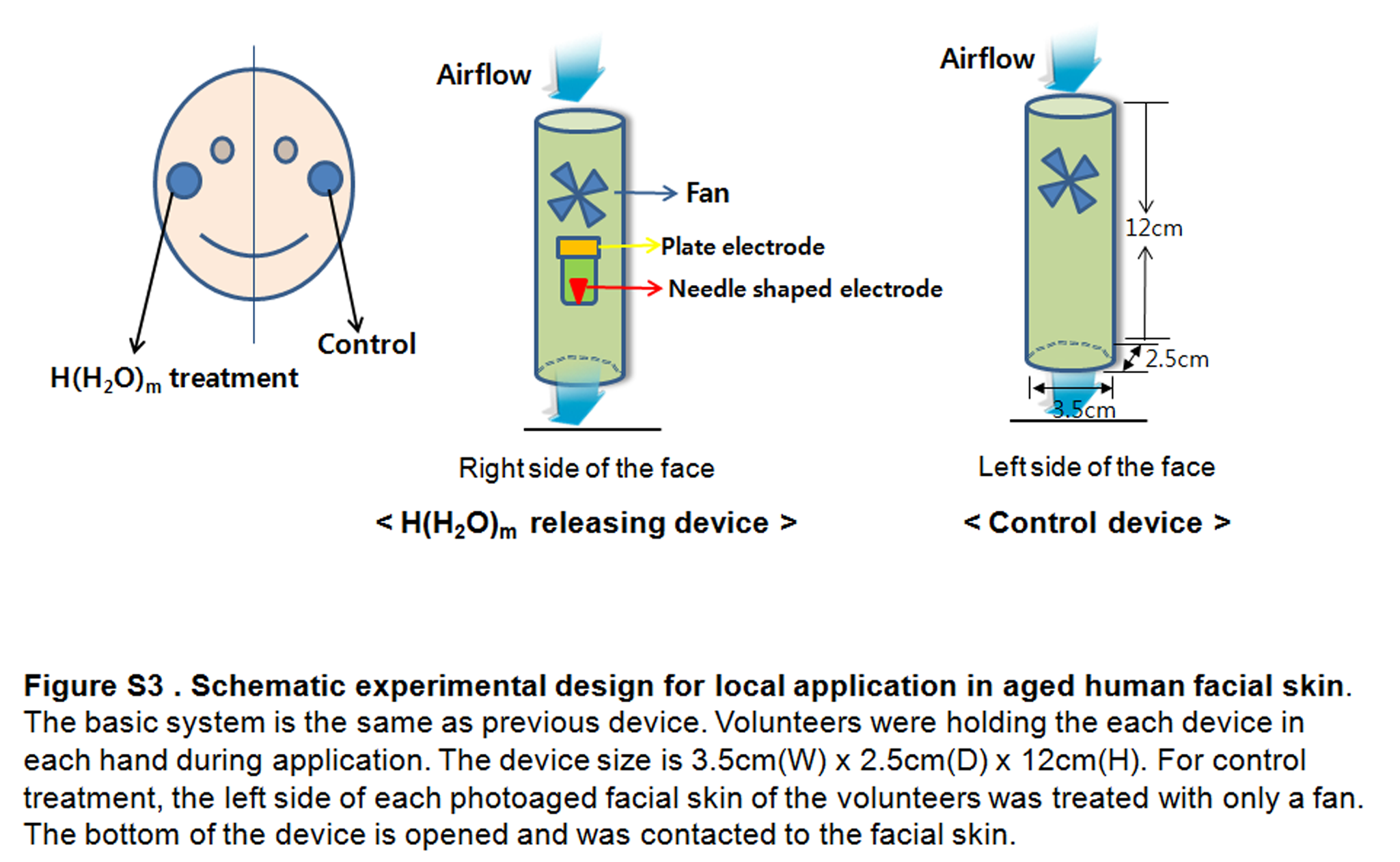

Supplement: Figure S3 — Schematic experimental design for local application in aged human facial skin. (TIF) [file pone.0061696.s003.tif]
